# Complement Activation and Regulation in Preeclamptic Placenta

**DOI:** 10.3389/fimmu.2014.00312

**Published:** 2014-07-09

**Authors:** Anna Inkeri Lokki, Jenni Heikkinen-Eloranta, Hanna Jarva, Terhi Saisto, Marja-Liisa Lokki, Hannele Laivuori, Seppo Meri

**Affiliations:** ^1^Department of Medical Genetics, Haartman Institute, University of Helsinki, Helsinki, Finland; ^2^Department of Bacteriology and Immunology, Haartman Institute, University of Helsinki, Helsinki, Finland; ^3^Immunobiology Research Program, Research Programs Unit, University of Helsinki, Helsinki, Finland; ^4^Department of Obstetrics and Gynaecology, Helsinki University Central Hospital, Helsinki, Finland; ^5^Division of Clinical Microbiology, Helsinki University Central Hospital Laboratory (HUSLAB), Helsinki, Finland; ^6^Transplantation Laboratory, Haartman Institute, University of Helsinki, Helsinki, Finland

**Keywords:** preeclampsia, complement, pregnancy, placenta, immunohistochemistry, innate immunity

## Abstract

Preeclampsia (PE) is a common disorder of pregnancy originating in the placenta. We examined whether excessive activation or poor regulation of the complement system at the maternal–fetal interface could contribute to the development of PE. Location and occurrence of complement components and regulators in placentae were analyzed. Cryostat sections of placentae were processed from 7 early-onset PE (diagnosis <34 weeks of gestation), 5 late-onset PE, 10 control pregnancies, and immunostained for 6 complement activators and 6 inhibitors. Fluorescence was quantified and compared between PE and control placentae. Gene copy numbers of complement components *C4A* and *C4B* were assessed by a quantitative PCR method. Maternal *C4* deficiencies (≥1 missing or non-functional *C4*) were most common in the early-onset PE group (71%), and more frequent in late-onset PE compared to healthy controls (60 vs. 38%). Complement C1q deposition differed significantly between control and patient groups: controls and early-onset PE patients had more C1q than late-onset PE patients (mean *p* = 0.01 and *p* = 0.005, respectively). C3 activation was analyzed by staining for C3b/iC3b and C3d. C3d was mostly specific to the basal syncytium and C3b/iC3b diffuse in other structures, but there were no clear differences between the study groups. Activated C4 and membrane-bound regulators CD55, CD46, and CD59 were observed abundantly in the syncytiotrophoblast. Syncytial knots, structures enriched in PE, stained specifically for the classical pathway inhibitor C4bp, whereas the key regulator alternative pathway, factor H (FH) showed a wider distribution in the placenta. Differences in C1q deposition between late- and early-onset PE groups may be indicative of the different etiology of PE symptoms in these patients. Irregular distribution of the complement regulators C4bp and FH in the PE placenta and a higher frequency of *C4A* deficiencies suggest a disturbed balance between complement activation and regulation in PE.

## Introduction

Preeclampsia (PE) is a serious complication of human pregnancy. It can lead to multi-organ dysfunction and, rarely, to a life-threatening convulsive condition, eclampsia (1). PE affects 3–5% of pregnancies in all ethnic groups. The development and progression of the disease are unpredictable. Presently, delivery of the placenta remains the only cure for PE. The etiology of PE is still largely unknown. Because of the unique challenge that the fetoplacental unit poses to the maternal immune system PE could involve a non-classical-type incompatibility (2).

The complement (C) system is a phylogenetically ancient means of self–non-self discrimination. It plays a central role in innate immune defense, clearance, and as a mediator of the adaptive immunity (3). Complement activation is regulated by soluble and membrane-bound inhibitor molecules. Activation of the C system releases potent anaphylatoxins, which generate inflammation by mediating chemotaxis, increased vascular permeability, smooth muscle contraction, and leukocyte activation. In addition, C has been indicated in the homeostatic clearance of waste products as well as in regenerative processes. The discrimination potential of C goes beyond that between self and non-self, because C can distinguish non-viable tissue components and cells from viable ones. In particular, a disturbed binding of the soluble regulator factor H (FH) to injured or altered host cells could allow an alternative pathway-mediated attack against such target. Also, exposed subcellular or disturbed membrane structures could initiate both classical and alternative pathway activation, either directly or via activating molecules like C-reactive protein (CRP) or natural antibodies.

Disturbances in C activity can predispose to infections or to a systemic lupus erythematosus (SLE)-like immunoinflammatory syndrome. The latter has been related to an inadequate waste disposal function of the classical pathway (4). These types of syndromes tend to become worse during pregnancy, possibly because of a greater challenge to the clearance system posed by material derived from the placenta. On the other hand, disturbances in C regulation can lead to such catastrophic consequences as the atypical hemolytic uremic syndrome (aHUS) and other forms of thrombotic microangiopathy (TMA) (5). Central to these is C attack against endogenous tissue structures, endothelial cells, and blood cells that can lead to vascular damage and organ failure, notably in kidneys. Pregnancy is a well-known potential trigger for such syndromes.

The depth of placentation required for a healthy human pregnancy presents a unique challenge to regulation of the maternal immunological processes (6, 7). For the trophoblast invasion and healthy placentation to occur, the fetal cells must avoid recognition by and activation of the C system (7). A risk for the latter exists because of the potential development of maternal antibodies or spontaneous activation of the C system by exposed villous structures, which are often observed in the PE placentae by microscopy.

A well regulated C system is a prerequisite for a healthy pregnancy (8, 9). Recently, mutations in C regulatory genes have been reported in women with recurrent pregnancy loss (10). Recently, a case report was published, where treatment with the C5 inhibitor eculizumab prolonged HELLP (hemolysis, elevated liver enzymes, and low platelets)/PE pregnancy by 17 days. The treatment resulted in a clinical improvement of the patient and normalization of her lab parameters supporting the role of C in the pathogenesis of PE (11). Up to our knowledge, however, the C system has never been described in its entirety in the PE placentae in comparison with healthy control placentae. In particular, little information is available on the possible role or dysfunction of the two major soluble inhibitors FH and C4b binding protein (C4bp) in the placenta during pregnancy.

**Figure 1:**
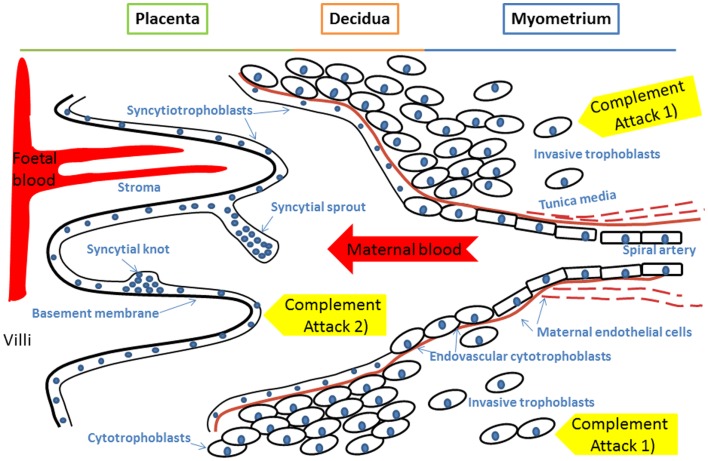
**A model of innate immunity incompatibility between maternal and fetal cells in preeclampsia and the maternal immune system**. Failure of complement regulation on fetal tissue or excessive activation of the maternal complement system could result in complement attack against 1) invading trophoblast cells or 2) placental syncytiotrophoblast that represent the discordant interfaces. Accordingly, an imbalance between complement activation and regulation could contribute to the pathogenesis of preeclampsia. Specific foci for complement to attach could include syncytial bodies (apoptotic syncytial knots and syncytial sprouts), which are observed more often in preeclamptic placentae than in healthy controls.

Two forms of *C4* exist, *C4A* and *C4B*, which are the targets for control by the C4bp regulator protein. Genes for *C4A, C4B*, as well as for C2 and factor B, are encoded in the MHC class III region in the human chromosome 6p21.3. *C4* genes are polymorphic, variations in gene copy numbers exist and deficiencies are common (12). Deficiencies in C2 and *C4A* predispose to SLE, a disorder known to worsen during pregnancy (13, 14). Because of the vascular disturbance in PE and its similarity to many diseases involving C dysfunction (SLE, phospholipid antibody syndrome, aHUS) we found it reasonable to hypothesize that an imbalance between C activation and regulation could be involved in PE (Figure 1).

To test the involvement of C in PE, we have analyzed immunohistochemically the deposition and expression of key activating components and regulators of the C system in preeclamptic placentae in relation to disease onset and in comparison to healthy placentae. The results favor the hypothesis that an insufficient complement function is linked to an inability to clear away trophoblast material from the placenta. As a consequence, the material deposits in fibrinoid clusters and could cause an endothelial–vascular disorder in the maternal circulation.

## Materials and Methods

### Patients

For this study, we chose randomly 12 women with PE and 10 controls without PE (Table 1) from the prospective arm of the Finnish Genetics of Preeclampsia Consortium (FINNPEC) cohort. While FINNPEC is a multicenter study, all women in this study delivered at the Helsinki University Central Hospital. Placental samples (nine-site biopsies) were collected after delivery from the patients. All pregnancies were singletons and exclusion criteria were multiple pregnancies or maternal age <18 years. An additional exclusion criterion was a known autoimmune disease such as SLE. All subjects provided a written informed consent and the FINNPEC study protocol was approved by the coordinating Ethics Committee of the Hospital District of Helsinki and Uusimaa.

**Table 1:** **Clinical characteristics of the study population**.

	Controls	Late-onset	Early-onset
	*N* = 10	PE, *n* = 5	PE, *n* = 7
Age	30.6 ± 3.1	33.8 ± 4.1	31 ± 6.5
Gravidity	1.8 ± 0.8	1.2 ± 0.5	1.9 ± 1.1
Parity	0.6 ± 0.8	0.2 ± 0.5	0.6 ± 1.0
Maternal BMI (kg/m^2^)	22.9 ± 2.7	21.0 ± 2.2	22.3 ± 2.4
Hypertension before pregnancy	1/10	1/5	2/7
Celiac disease	0/10	1/5	0/0
Thrombophilia	0/10	0/5	1/7
PE in previous pregnancy	0/10	1/5	1/7
Early pregnancy systolic BP (mmHg)	114 ± 8	117 ± 7	129 ± 10^b^^,^^c^
Early pregnancy diastolic BP (mmHg)	73 ± 8	77 ± 5	82 ± 9^a^
Highest systolic BP (mmHg)	128 ± 13	166 ± 10^b^	167 ± 16^b^
Highest diastolic BP (mmHg)	87 ± 10	109 ± 8^b^	118 ± 9^b^
Highest proteinuria (g/24 h)	–	1.8 ± 0.4	5.7 ± 3.8^b^^,^^c^
Gestational weeks at birth	40 ± 2	38 ± 2^a^	33 ± 4^b^^,^^c^
Birth weight (g)	3646 ± 282	2938 ± 423^b^	1842 ± 544^b^^,^^d^
Complications
IUGR	–	–	3/7
Placental insufficiency	–	–	2/7
HELLP	–	1/5	1/5

*^a^Significant in <0.05 level when compared with controls*.

*^b^Significant in <0.01 level when compared with controls*.

*^c^Significant in <0.05 level when compared with late-onset group*.

*^d^Significant in <0.01 level when compared with late-onset group*.

*Mean ± SD values are shown*.

*PE, preeclampsia; BMI, body mass index; BP, blood pressure; IUGR, intrauterine growth restriction; HELLP, hemolysis; elevated liver enzymes, low platelets*.

Preeclampsia was defined as hypertension and new-onset proteinuria occurring after 20 weeks of gestation. Hypertension was defined as systolic blood pressure of 140 mmHg or more, and/or a diastolic blood pressure of 90 mmHg or more after 20 weeks of gestation. Proteinuria was defined as the urinary excretion of ≥0.3 g protein in a 24-h specimen, or 0.3 g/l or, in the absence of concurrent quantitative measurement, at least a “2+” or more, or two “1+” proteinuria dipstick readings with no evidence of urinary tract infection. PE was considered severe if blood pressure was ≥160/110 mmHg, or proteinuria exceeded 5 g/24 h, or symptoms like cerebral or visual disturbances or abdominal pain appeared. Intrauterine growth restriction (IUGR)/placental insufficiency was defined as birth weight below −2SD and/or umbilical artery resistance ≥+2SD according to gestational age specific standards (15) without known etiology unrelated to the aims of the present project (e.g., congenital malformation syndromes and chromosomal defects). We have divided the PE women into two groups according to the weeks of gestation at diagnosis: early-onset <34 weeks of gestation (*n* = 7), late-onset ≥34 weeks of gestation (*n* = 5).

Placental samples were chosen preferentially from patients with severe and early-onset PE. Chronic hypertension (an elevated blood pressure that predated the pregnancy or detected before mid-pregnancy) was observed in three PE women and one woman in the control group (Table 1). Two women had HELLP syndrome. One woman of the late-onset PE group had celiac disease and one patient of the early-onset PE group had thrombophilia caused by mutation in the coagulation factor FII.

### Sample preparation

Approximately 1 cm wide tissue samples from the placentae were dissected using a scalpel and scissors and placed in a cryotube for preservation. Following the nine-site procedure, the placenta was visually divided into nine pre-specified regions and one sample was taken from each region. Within 2 h of the delivery of the placenta, the cryotubes containing samples were placed into the inner compartment of a nested metal holder. Approximately 150 ml of liquid nitrogen was added to the outer compartment to cool down the 150 ml isopropanol poured into the inner compartment. According to a standardized tissue-preserving collection procedure, the samples were left for 20 min to freeze slowly through the isopropanol pool. When isopropanol reached a floury frozen state, the cryotubes were stored at −80°C. For our study, one region (no. 5) was immunohistochemically analyzed from all samples.

### Antibodies against complement factors

The antibodies used are listed in Table 2. Primary antibodies were chosen to detect either the activating components or regulators of the alternative and classical pathways of complement activation. Soluble endoglin (s-eng) was used as a positive control for changes in PE placenta (16).

**Table 2:** **Primary antibodies used for immunofluorescence stainings**.

Antibody	Type	Dilution	Source^a^	Role
C1q	Rabbit pAb	1:1000	DAKO	CP component
C4c	Rabbit pAb	1:400	DAKO	CP component
C4bp	Sheep pAb	1:200	The Binding Site	CP regulator
CRP	Mouse mAb	1 μg/ml	Fitzgerald	CP activator
C3c	Rabbit pAb	1:1000	DAKO	AP component
C3d	Rabbit pAb	1:1000	DAKO	AP component
Factor H	Goat pAb	1:400	Calbiochem	AP regulator
C9	Goat pAb	1:400	Quidel	TP component
MCP (CD46)	Mouse mAb	1 μg/ml	IBGRL	AP and CP regulator
Bric 230 (CD55)	Mouse mAb	1 μg/ml	IBGRL	AP and CP regulator
Bric 229 (CD59)	Mouse mAb	1:200	IBGRL	TP regulator
CR1	Mouse mAb	1 μg/ml	AbD Serotec	AP and CP regulator
s-Endoglin	Mouse mAb	2 μg/ml	Santa Cruz	PE indicator

*^a^DAKO, Glostrup, Denmark; The Binding Site, Birmingham, UK; Fitzgerald Industries International, North Acton, MA, USA; Calbiochem, Merck KGaA, Darmstadt, Germany; Quidel Corporation, San Diego, CA, USA; IBGRL The International Blood Group Reference Laboratory, Bristol, UK; AbD Serotec, Oxford, UK; Santa Cruz Biotechnology, Inc., Dallas, TX, USA*.

*pAb, polyclonal antibody; mAb, monoclonal antibody*.

*CP, classical pathway; AP, alternative pathway, TP, terminal pathway; PE, preeclampsia*.

Anti-C3c antibody was used to detect C3b and iC3b, which are the products of alternative pathway activation and amplification and of subsequent C3b inactivation. C3d fragment was separately stained for because the C3d antibody recognizes the C3dg fragment, which remains surface bound after the release of C3c. FH binds to the C3b molecule on the self-cell surface, where it can be detected by the FH antibody. The C1q antibody recognizes several different structures of the classical pathway activating C1q molecule. The C4c antibody recognizes the native C4 molecule (both C4A and C4B) as well as the activation product C4b and its inactivated form iC4b. For detection of C4bp, a cofactor for C4b in activation, a polyclonal sheep antibody was used. Membrane-bound DAF (CD55) and MCP (CD46) were analyzed by specific mouse monoclonal antibodies.

### Immunofluorescence staining

The frozen tissue samples were cryosectioned at 5 μm and when possible two or three serial sections were laid per each slide. The dried sections were rinsed with phosphate-buffered saline (PBS) and moist samples were blocked against non-specific binding with 1% bovine serum albumin (BSA) in PBS for 15 min in a humid chamber. Excess liquid was discarded and the first antibody was pipetted to the sample in 1% BSA/PBS as detailed in Table 2. One section on the slide was used for a mock staining by treating it with only 1% BSA/PBS without the first antibody (Figures 4D,H,L; 5D,H and 6D,H,L). After washing for five times (1 min each) the second antibody was pipetted at a 1:300 dilution in 1% BSA/PBS. Second antibodies used were Alexa 488-labeled antibodies against goat, rabbit, mouse, and sheep immunoglobulins (Invitrogen, Thermo Fisher Scientific, Waltham, MA, USA). After 20-min incubation and five 1-min rinses, excess liquid was removed and a mounting medium was added. Stained sections were kept at +4°C in the dark until documentation on the same day.

### Histochemistry

One slide from each sample was used for standard automated hematoxylin and eosin (HE) staining to ensure diagnostic-level consistency and to obtain a histological reference point for the immunofluorescence (IFL) analyses.

### Imaging and histological analysis

Data were collected using standardized fluorescence microscopy settings, where all slides were photographed with 10×, 20×, and 40× magnifying objectives. Exposure times per each magnification were 20, 50, and 83.3 ms. Images were collected using Olympus DP Manager (ver. 2.2.1.195) and Olympus DP Controller (ver. 2.2.1.227) image capture softwares with Olympus BX51 fluorescence microscope camera.

The same protocol and machinery for imaging was used for histochemistry preparations. Images captured from HE stained samples were used to identify key structures and to characterize the placenta. The structures to be identified included stem villi, small villi, villous fibrinoid (i.e., as a part of a villus, often replacing syncytium), fibrinoid necrosis, syncytiotrophoblast (STB), cytotrophoblast, fetal arterial endothelium, and syncytial bodies (incl. syncytial sprouts and syncytial knots). The occurrence of syncytial bodies and fibrinoid structures was semiquantified by grading (0 = none observed, 1 = counted 1–3, 2 = >3, 3 = all over, cannot be counted). Structural integrity was measured by intactness of syncytium and special attention was paid on shedding of syncytial cells. Signs of nuclei of apoptotic cells were looked for and noted.

### Genetic analysis

To correlate the C4 deposition observed in the placentae with the functional *C4* genes, *C4A* and *C4B* gene copy numbers and a silencing *C4A* mutation were analyzed using a previously published protocol (12). Briefly, a SYBR^®^ Green labeled real-time quantitative polymerase chain reaction (qPCR) with a specified concentration range approach was used to obtain numbers of *C4* and to detect deficiencies due to CTins, which renders the affected *C4A* non-functional. Two copies of *C4A* and *C4B* are considered the normal genotype and while deviations from the four-gene norm are common, individuals with less than two genes for either *C4* gene or individuals with *C4A* CTins mutation are considered *C4* deficient. DNA for the qPCR protocol was extracted from whole blood samples of mothers and from umbilical cord blood samples collected post-partum from the placenta. Blood samples were stored in −80°C and DNA was later extracted using Macherey-Nagel NucleoSpin Blood XL kit (Macherey-Nagel GmbH & Co., KG Düren, Germany). Extracted DNA was stored at −80°C until used in the analysis.

### Statistical analysis

**Figure 2:**
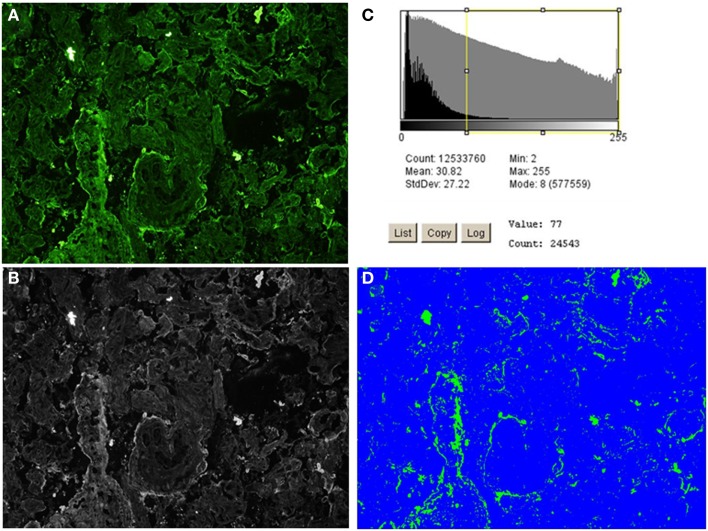
**High-intensity analysis workflow of C4bp staining of an early-onset preeclamptic placenta using ImageJ 1.46 software**. The image is processed through steps **(A–D)** to produce a quantification of the high-intensity fluorescence areas, which correspond to the structures where **(C)** deposition/expression is most conspicuous. **(A)** The original image. **(B)** Black and white rendering of the image in **(A)**. **(C)** Threshold set at 75% positive fluorescence (calculated to be value 77 for this image). **(D)** Area (in pixels) of positive signal (in green), % area is given in output and compared across and between the patient groups.

ImageJ 1.46 and Fiji-win32 softwares were used to quantify the intensity of fluorescence in the fixed magnification images. These were chosen to minimize the variation of staining quality and tissue quality between individuals, which was more apparent at the highest levels of magnification. To correct for false positive readings resulting from background autofluorescence, mean intensity +1 SD $\left(\bar{X}+\sigma \right)$ was determined to be 7 at 20 ms exposure and 15 at 50 ms exposure. This was calculated from analysis of negative controls (Figures 4D,H,L; 5D,H and 6D,H,L). Using the appropriate zero thresholds each image was analyzed for several parameters of fluorescence intensity. Sum was defined as mean intensity * area of positive fluorescence in pixels $\left(\bar{X}\times \sigma \right)$. The purpose of using different parameters was to differentiate between different patterns as well as intensity of fluorescence. Log_10_ transformation was used to normalize the image capture data and normality of the transformed data was verified by Shapiro–Wilk *W* test (data not shown). An independent-samples *t*-test for analyzing the significance of differences between means of values obtained from patient groups and controls was carried out for key statistical parameters including sum and mean as well as the clinical measurements. For the high-intensity analysis, the top 75% proportion of fluorescence histogram was determined for each image using ImageJ 1.46 software and the calculated maximum fluorescence value (Figure 2). A filter was placed at the calculated 75% minimum value creating representative images of high-intensity regions. Statistical testing of the high-intensity area percentage was done as above (data not shown). Furthermore, Pearson-correlation of high-intensity proportions between different stainings was calculated for each patient group independently. Positive correlations were used as an indicator of two components observed in the same sample, while negative correlations were interpreted as two components occurring in different samples. Fisher’s exact test was used to analyze for differences between segregation of *C4* gene deficiencies between groups of patients, and independent-samples *t*-test was used to assess the association of *C4* gene deficiencies and immunohistochemistry fluorescence sum and mean values.

## Results

**Figure 3:**
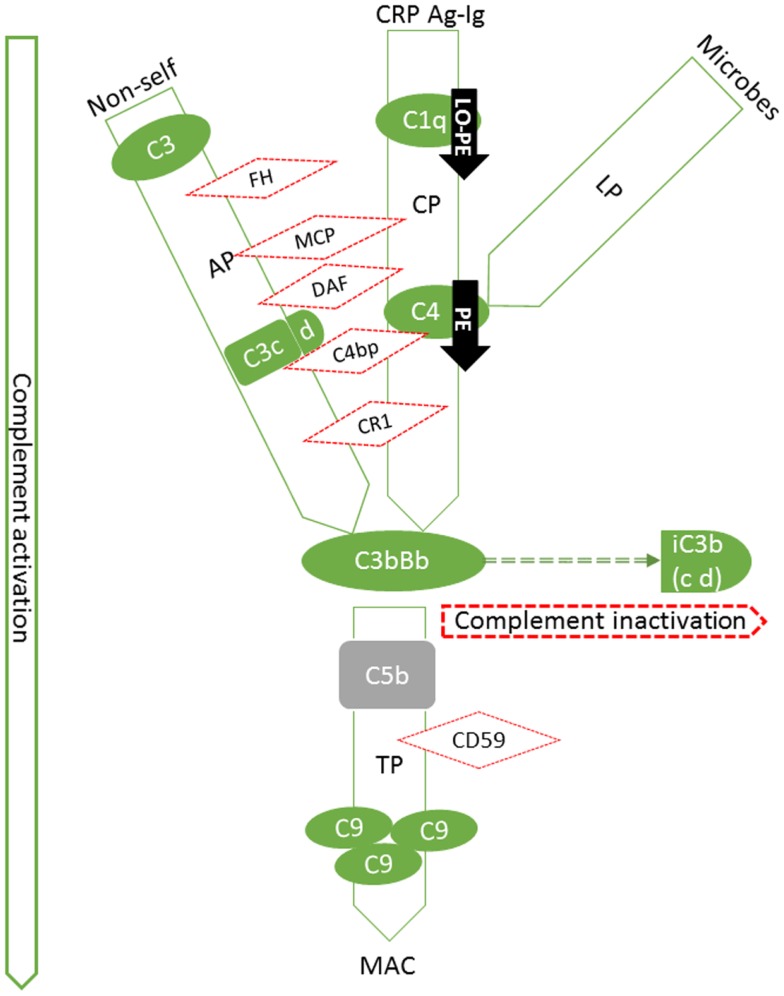
**Summary of expression patterns of complement components in the placenta**. Our findings are highlighted in black arrows (PE: preeclampsia, LO-PE: late-onset preeclampsia). Pictured are the components that we studied in their respective position in the activation cascade (green solid lines and shapes) or regulatory network (red dotted lines and shapes). The C system is composed of approximately 20 plasma proteins, which can be activated in a stepwise cascade via the classical (CP, via Ag-Ig: immunocomplexes or CRP: C-reactive protein), lectin (LP), or the alternative pathway (AP). In addition, there are approximately 15 components that act as receptors or protective molecules on cell membranes. All three pathways result in the activation of the main complement component C3 and thereafter of the terminal pathway causing the formation of membrane attack complexes (MACs) and ultimately target cell damage. C5b (not studied, in gray) is the activated component at the onset of terminal pathway. FH is a regulator of the alternative pathway, where its main role is to act as a cofactor for Factor I in the cleavage of C3b into iC3b. Similarly in the classical pathway, cleavage of C4 to activated form C4b is inhibited by a potent regulator C4bp. C3b and C4b are both generated by activation of not only the classical, but of the lectin pathway as well. C1q is a potent activator of the classical pathway. Binding of the complex (C1qr_2_s_2_) activates the C4 step. Together with decay accelerating factor (DAF; CD55) and membrane cofactor protein (MCP, CD46), C4bp regulates the progression of the classical C pathway by controlling the formation and function of the classical pathway C3 convertase, C4b2a. Like FH, MCP can also act as a cofactor in C3b inactivation. In the classical pathway, DAF accelerates the disassembly of C4b2a and in the alternative pathway that of C3bBb. DAF is a glycosylphosphatidylinositol-anchored membrane molecule. Complement receptor type 1(CR1, CD35) is a membrane-bound regulator expressed primarily by bone-marrow derived cells.

Activating components and regulators of the C system as well as s-endoglin were found to be deposited in the placenta in a structure-specific manner showing differences between patient groups and controls. In the following, the results are presented according to C pathways (Figure 3).

### Classical pathway

#### C1q

**Figure 4:**
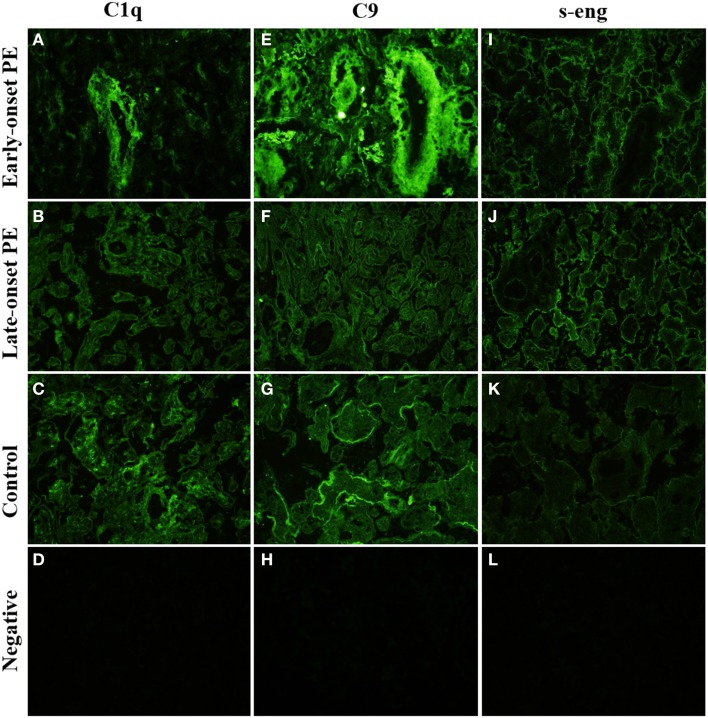
**Side-by-side comparison of C1q, C9, and s-Eng staining of the same individual placentas**. C1q is absent from large villi of the late-onset PE control. **(B)** There is a negative correlation between high-intensity areas of C1q and C9. s-Endoglin is deposited on the syncytiotrophoblast of PE placentae **(I,J)** and absent from the control placenta **(K)**. Top row **(A,E,I)**: early-onset PE. Middle top row **(B,F,J)**: late-onset PE. Middle bottom row **(C,G,K)**: control. Bottom row **(D,H,L)**: negative control (antibody I omitted). 20× magnification.

C1q was observed at the STB layer in 5/10 controls but in none of the early- onset PE patients and only in one of the late-onset PE patients. Overall, we found less C1q in the PE patients when compared with normal controls (Figures 4A–C). In about half of the cases, C1q was observed in the endothelia of placental vessels. In the ImageJ analysis, the amount of C1q deposition was higher in controls and early-onset PE group than in late-onset PE group (mean *p* = 0.01 and mean *p* = 0.005, respectively) (Tables 4 and 5). Accordingly, the area of high-intensity regions for C1q staining was significantly smaller in the late-onset PE cases than in controls (*p* = 0.005) and in the early-onset PE group (*p* = 0.011). In the areas of high-intensity staining, the tissue structure was breaking down suggesting an on-going necrotic process. C1q was frequently found in the stromal areas of the larger villi. Samples with large villi containing C1q abundantly were observed more frequently in the patients (both groups combined) vs. controls (58 vs. 40%), especially in the early-onset PE group (71%). C1q was present also in the fibrinoid necrotic areas especially in the placentae of patients in the early-onset PE group. Less C1q was seen in the fibrinoid areas of the late-onset group or normal controls.

#### C4 gene numbers

*C4A* or *C4B* deficiencies were found almost twice as often in early-onset PE patients than in healthy controls (Table 3). *C4A* deficiencies were found in 40% (2/5) of PE mothers with late-onset disease and 43% (3/7) of PE mothers with early-onset disease. None were observed in the controls (*n* = 7). Because of the small number of samples, the difference was on the borderline statistically significant (*p* = 0.055 Fisher’s exact two-sided test). Only two individuals had total *C4* deficiency (total *C4A* deficiency in one control mother and total *C4B* deficiency in one child born from early-onset PE pregnancy).

**Table 3:** **Frequency of *C4A* and/or *C4B* deficiencies in preeclamptic (PE) patients and controls**.

	C4A or C4B deficiency		C4A deficiency		C4B deficiency	
	Maternal	Fetal	Maternal	Fetal	Maternal	Fetal
PE (pooled) (*n* = 12)	0.667	0.700	0.417	0.300	0.333	0.400
Early-onset PE (7)	0.714	0.667	0.429	0.333	0.286	0.333
Late-onset PE (5)	0.600	0.750	0.400	0.250	0.400	0.500
Control (8)	0.375	0.500	0	0.375	0.375	0.250

#### C4

**Figure 5:**
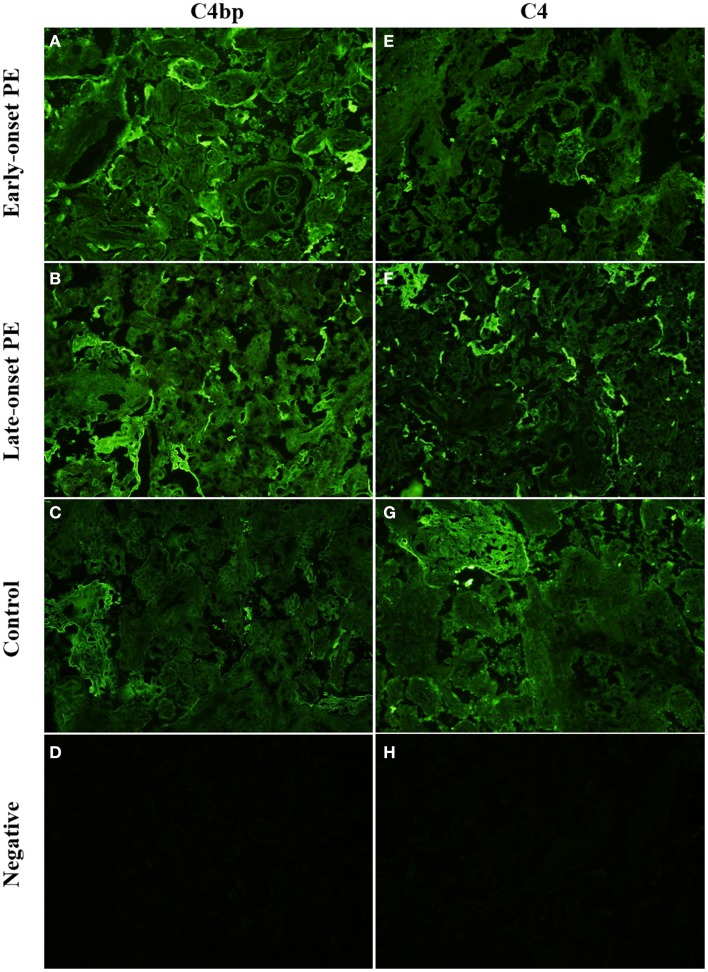
**Side-by-side comparison of C4 and C4bp stainings in the same placental samples**. C4 is deposited in syncytial knots, visible as bright clusters but also in the syncytia of selected villi. C4bp is observed in syncytial bodies. C4bp is not observed in a circumferential pattern of the syncytium. C4 and C4bp are both observed in fibrinoid structures [here in control panels **(G,C)**]. Top row **(A,E)**: early-onset PE. Middle top row **(B,F)**: late-onset PE. Middle bottom row **(C,G)**: control. Bottom row **(D,H)**: negative control (antibody I omitted). 20× magnification.

C4 deposits were observed mainly in the STB layer, either in the apical membrane or throughout the syncytium. There was no clear difference between the patient groups and controls in this pattern (Tables 4 and 5). A particular staining for C4 was seen in clusters formed from the STB layer. These clusters represent syncytial bodies (i.e., syncytial knots or sprouts) and shedding of the syncytium. The number of C4 clusters was slightly higher in the preeclamptics (Figures 5E–G). The intensity of the C4 fluorescence mean or sum values did not associate with *C4A* or *C4B* deficiencies of the mother or fetus.

**Table 4:** **Complement and s-endoglin deposition in early- and late-onset preeclampsia (PE) vs. control**.

	Type of PE onset	C3b/iC3b			C1q			C4bp			C4		
		Control			Control			Control			Control		
		*t*	*p*	df	*t*	*p*	df	*t*	*p*	df	*t*	*p*	df
Sum	Late	1.735	0.106	13	**2.793**	**0.015**	**13**	−0.487	0.635	13	0.969	0.352	12
	Early	−0.671	0.512	15	−0.530	0.604	15	−0.667	0.516	14	0.486	0.635	14
Mean	Late	1.718	0.110	13	**2.993**	**0.01**	**13**	−0.374	0.714	13	0.703	0.495	12
	Early	−0.607	0.553	15	−0.091	0.929	15	−0.507	0.620	14	0.432	0.672	14

	**Type of PE onset**	**FH**			**C3d**			**C9**			**s-Endoglin**		
		**Control**			**Control**			**Control**			**Control**		
		***t***	***p***	**df**	***t***	***p***	**df**	***t***	***p***	**df**	***t***	***p***	**df**

Sum	Late	0.059	0.954	13	−0.217^a^	0.836	5.463	0.825	0.424	13	*1.842*	*0.090*	*12*
	Early	−0.913^a^	0.378	12.533	−0.365^a^	0.723	9.333	−0.129	0.899	15	1.524	0.150	14
Mean	Late	0.067	0.947	13	0.097	0.924	13	0.722	0.454	13	**2.825**	**0.015**	**12**
	Early	−0.812^a^	0.431	12.927	−0.009	0.993	15	−0.215	0.833	15	*1.815*	*0.091*	*14*

*Mean fluorescence intensity and sum of intensity are compared between early- and late-onset PE and controls. C1q and soluble endoglin show significant or borderline significant differences*.

*^a^Equal variances not assumed*.

*Bold significant to the 0.05 level*.

*Italics borderline significant*.

*df, degrees of freedom*.

**Table 5:** **Complement and s-endoglin deposition in early- vs. late-onset preeclampsia (PE)**.

	Type of PE onset	C3b/iC3b			C1q			C4bp			C4		
		Late			Late			Late			Late		
		*t*	*p*	df	*t*	*p*	df	*t*	*p*	df	*t*	*p*	df
Sum	Early	1.219	0.251	10	**2.273**	**0.046**	**10**	0.176	0.864	9	0.639	0.537	10
Mean	Early	1.297	0.224	10	**3.614**	**0.005**	**10**	0.127	0.902	9	0.364	0.723	10

	**Type of PE onset**	**FH**			**C3d**			**C9**			**s-Endoglin**		
		**Late**			**Late**			**Late**			**Late**		
		***t***	***p***	**df**	***t***	***p***	**df**	***t***	***p***	**df**	***t***	***p***	**df**

Sum	Early	0.773	0.457	10	0.074	0.943	10	0.857	0.412	10	0.588	0.569	10
Mean	Early	0.722	0.487	10	0.085	0.934	10	0.810	0.437	10	0.511	0.621	10

*Mean fluorescence intensity and sum of intensity are compared between early- and late-onset PE and controls. Both sum and mean of C1q differs significantly between the two patient groups*.

*Bold significant to the 0.05 level*.

*df, degrees of freedom*.

#### C4bp

C4bp is an inhibitor of the classical pathway occurring usually physiologically in complex with the anticoagulant protein S. Overall, C4bp was found deposited particularly in small syncytial bodies, which appeared as brightly staining particles attached or sometimes shed from the syncytium (see Figures 1 and 5B). Characteristically, these were seen as dense clusters of bright staining, which were interpreted as small necrotic, apoptotic, or fibrinoid tissue fragments, typically syncytial knots (Figure 5A). In contrast to other C regulators C4bp was not deposited in a circumferential continuum on the syncytium, while it was typically observed only on the apical surface of the syncytium. Of the controls 80% (8/10) showed apical C4bp staining in the STB whereas in PE cases 42% (5/12) had some C4bp deposition on the STB layer (Figure 5). The stromas of the villi were negative and also the placental endothelium was mostly negative for C4bp. In a few placentae small necrotic/fibrin containing areas were intensely stained for C4bp (Figure 5C). These were observed in both cases and controls.

C-reactive protein and Complement Receptor type 1 (CR1; CD35) were tested with four samples representing one early-onset PE, two late-onset PE and one control specimens. Both staining were negative. CRP and CD35 were subsequently omitted from the protocol.

### Alternative pathway

#### C3

**Figure 6:**
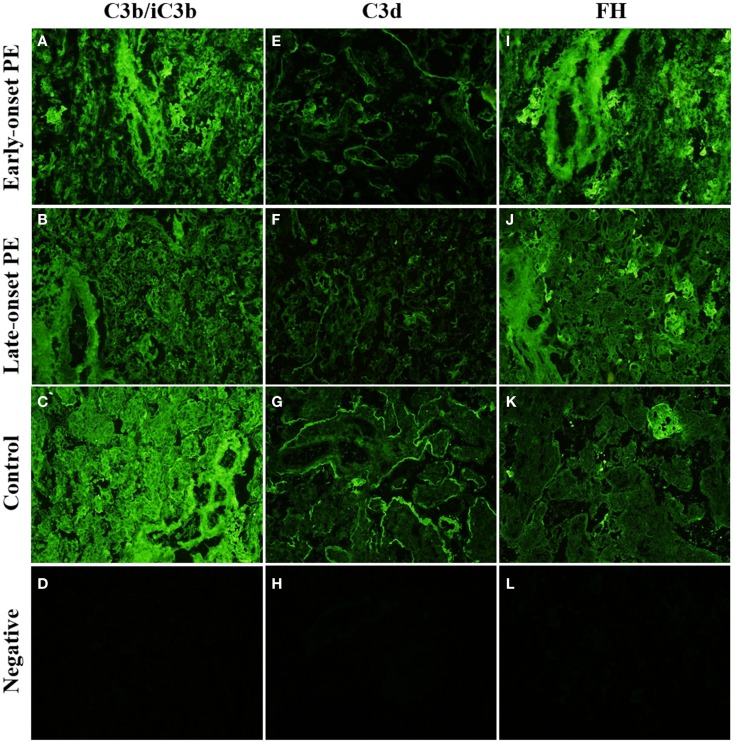
**Side-by-side comparison of C3b/iC3b, C3d, and FH staining patterns in the same placentas**. C3b/iC3b and FH are observed in stromas of large stem villi [early-onset preeclampsia (PE): **(A,I)** and control: **(C)**] and in fibrinoid structures **(J)**. C3d is typically observed in circumferential pattern of basal membrane of the syncytium **(E–G)**. The circumferential pattern is often interrupted in PE **(E,F)** where physical damage to the syncytium causes increased rate of syncytial shedding. Top row **(A,E,I)**: early-onset PE. Middle top row **(B,F,J)**: late-onset PE. Middle bottom row **(C,G,K)**: control. Bottom row **(D,H,L)**: negative control (antibody I omitted). 20× magnification.

In general, C3 (C3b, iC3b) detected by an antibody against C3c was abundantly present in the placenta. It was found in the STB layer and in the villous stroma (Figures 6A–C). In 4 out of 10 control placentae (40%) C3 reactivity was observed on the apical and basal linings of the STB layer. In contrast, this was observed only in one out of seven samples (14%) of the early-onset PE group. In half of all samples weak staining for C3 was also observed in the placental endothelium, without clear differences between the study groups.

The intensity of C3 deposition appeared weaker in the PE group and even more so in the late-onset group when compared with normal pregnancy (Figures 6B,C).

The samples were stained separately also for C3d because it is the C3 activation product that remains covalently bound in areas of C3b deposition thus reflecting a longer period of C activation. C3d deposition was also present in the placenta (Figures 6E–G). Reactivity was seen in the villous stroma but a clearer pattern was observed in the syncytium. C3d reactivity was observed mainly in the basal membrane of the syncytium (14/22). The pattern on the syncytial basement membrane was either partially or more fully circumferential lining of the villi (Figure 6G). No clear difference was observed between the patient groups and normal controls in the C3d abundance or pattern (Tables 4 and 6).

**Table 6:** **Complement and s-endoglin deposition in preeclampsia (PE) vs. control**.

	dg	C3b/iC3b			C1q			C4bp			C4		
		Control			Control			Control			Control		
		*t*	*p*	df	*t*	*p*	df	*t*	*p*	df	*t*	*p*	df
Sum	PE	−1.417	−0.172	20	−1.695	0.106	20	−0.775	0.448	19	0.848	0.407	19
Mean	PE	−1.366	0.187	20	−1.493	0.151	20	−0.602	0.554	19	−0.664	0.515	19

	**dg**	**FH**			**C3d**			**C9**			**s-Endoglin**		
		**Control**			**Control**			**Control**			**Control**		
		***t***	***p***	**df**	***t***	***p***	**df**	***t***	***p***	**df**	***t***	***p***	**df**

Sum	PE	0.458	0.652	20	0.045	0.964	20	−0.411	0.685	20	*2.008*	*0.059*	*19*
Mean	PE	0.407	0.689	20	−0.398^a^	0.695	18.961	−0.330	0.745	20	**2.463**	**0.023**	**19**

*Pooled PE and controls compared using mean fluorescence intensity and sum of intensity as measurements. Only soluble endoglin is significantly (mean) or borderline (sum) significantly different across the groups*.

*^a^Equal variances not assumed*.

*Bold significant to the 0.05 level*.

*Italics borderline significant*.

*df, degrees of freedom*.

*dg, diagnosis*.

#### Factor H

Factor H is a key regulator of the C amplification cascade in the alternative pathway. There was significant variation in the FH staining patterns in different placentae (Figures 6I–K). In the control placentae, FH localized evenly to the STB layer in 70% (7/10) of the placentae. Often both the apical and the basal layer were stained. Apical side of the STB layer was sometimes more strongly or exclusively stained.

In certain PE placentae, only the STB layer of some villi and fibrin clusters had become strongly stained for FH, while the rest of the tissue remained negative (Figure 6K). In other samples, villus stroma was FH-positive throughout, with distinct patterns in the fetal endothelium, STB layer, and in fibrin clusters (Figure 6I). The basal–apical STB layer pattern observed in most controls was only observed in a third (4/12) of the PE placentae. C1q depositions correlated negatively with the soluble regulator FH in the late-onset group (Table 7). Calcareous and necrotic areas were intensely stained for FH (Figure 6K).

**Table 7:** **Correlations of high-intensity area percentage values between different components per patient group**.

	Component 1	Component 2	Pearson’s *r*	*p* (2-Tailed)
Early-onset PE	**C1q**	**C9**	*****−***0.878**	**0.009**
	DAF	C4	0.824	0.023
	C3b/iC3b	s-Eng	0.829	0.021
Late-onset PE	C1q	FH	**−**0.932	0.021
	C4	C9	0.921	0.026
	**C4bp**	**C4**	** 0.962**	**0.009**
Control	DAF	MCP	0.634	0.049
	C4bp	MCP	−0.659	0.038
	FH	C9	0.671	0.034

*Significant correlations to the 0.01 level is indicated in bold, others are significant to 0.05 level*.

*PE, preeclampsia*.

### Terminal pathway

The terminal complex of C activation was assessed by staining for tissue associated C9. It could represent deposited polymeric MAC or tissue-bound SC5b-9 complexes. Positive staining for C9 was found in distinct regions of the placentae (Figures 4E–G). A strong staining was seen in the basal membrane of the STB layer (Figure 4G). C9 was also seen in the villous stroma in a non-regular pattern (Figure 4E). The most abundant staining for C9 was seen in the fibrinoid and necrotic/calcified areas. There were no differences between the PE and control groups in the C9 staining patterns or intensities (Tables 4–6).

### Membrane regulators

The membrane-bound regulators of complement MCP, DAF, and CD59 were typically observed in a dual pattern, where both the apical and the basal layer of the STB stained positive. Membrane regulators were mainly observed in the STB layer and to a lesser extent in the villar endothelium. DAF showed the weakest stainings of the membrane regulators. Unlike the soluble regulators, membrane-bound regulators were not observed in the syncytial bodies (Figure 1) nor in apoptotic, necrotic, or fibrinoid structures. No differences in the expression patterns of membrane regulators were observed between PE patients and healthy controls or between the early-onset and late-onset PE (data not shown). The C3b/C4b receptor CR1 (CD35) was not detected in the placentae of the control group or patients (data not shown). This indicates the lack of leukocytes in the placenta.

### s-Endoglin

We found s-endoglin to be significantly more abundant in PE pregnancies than in healthy controls (Table 6), especially in the late-onset disease group (Tables 4 and 5).

In PE, villi in 83% (10/12) of the placentae had distinct and circumferential endoglin deposition on the apical sides of the STB, sometimes penetrating through to the basal layer. Villi with negative staining in the STB were scarce (Figures 4I,J). In control placentas, s-endoglin was found mostly in the apical side of the STB layer. 20% of controls had a thin but circumferential deposition pattern on the STB of the villi. While some villi displayed a somewhat even positive layer, many villi stained negative or had only a few faintly positive deposits (Figure 4K).

## Discussion

Differences between women with and without PE were seen in the classical pathway of C activation and in the binding of protective C regulators to the placental structures. The critical differences were mostly observed at the STB layer and in lesions representing injured tissue structures. C1q deposition on the STB was least abundant in the late-onset PE group. Interestingly, in our small study sample we observed that *C4* deficiencies were more common in women with PE compared to women without PE. C4bp was localized to the syncytial bodies, which were more commonly seen in PE. FH was seen in the villi around the STB, although the extent of its binding varied greatly between individuals. In PE patients, less FH in the STB was observed.

Abundant evidence suggests that immunological mechanisms are involved in the various steps of PE pathogenesis. These include an incomplete spiral artery remodeling that causes poor placental development and creates turbulent and constrained blood flow to the villi (17). This in turn will aggravate the physical strain on the placental tissue and may lead to vascular symptoms typical for PE. An abnormal C function could limit the ability of C to maintain its waste disposal function. Accumulation of waste products in the placenta and insufficient repair functions could be related to the pathophysiology of PE.

The overall deposition of C1q was stronger in the early-onset than in the late-onset PE, which may reflect the difference in the etiopathogenesis between these two patient groups. Placental dysfunction is typically observed in the early-onset disease. We observed a higher frequency of necrotic large villi and an increased number of fibrinoid necrotic areas in the early-onset PE placentas (2, 18, 19). Importantly, in the early-onset PE placentae, a high-intensity area of C1q was found to lack C9, which indicates that C1q deposition probably does not result in the activation of the terminal pathway in these patients. If C4 deposition is considered indicative of classical pathway activation, missing C1q in the PE patients’ syncytium, where C4 deposits were typically observed, suggests that C1q deposition has other functions apart from classical pathway activation in the placenta. The classical pathway of the C system is triggered e.g., by the binding of C1q to immune complexes, CRP and other pentraxins or by intracellular components released upon cell damage (20). The possibly elevated plasma CRP levels of PE patients were not reflected in the placental deposition of CRP according to our findings (21).

Our results support the theory, that C1q has an important role in the maintenance of immune tolerance by clearing apoptotic and self-antigens. C1q has an important ability to recognize altered or exposed structures of self thereby leading to their efficient clearance by phagocytes without lysis and inflammation (22). Direct binding of C1q may occur to various structures, such as to phospholipids or vimentin exposed by vascular endothelia during tissue damage (23). Deficiency in C1q is associated with a major insufficiency in the clearance of apoptotic cells. This causes an SLE-like disease often involving glomerulonephritis (24). In PE, a partially similar function for C1q could be envisioned. Continuing stress and, by definition, the temporary existence of placenta may predispose the STB to cellular damage.

High-intensity areas of C1q deposition were negatively correlated with the soluble regulator FH in the late-onset PE group. This could be a reflection of a C1q/FH balance. Our results suggest that C1q could also bind to structures exposed by a turbulent blood flow during PE. These structures could be within the connective tissue, in vascular endothelia or on the trophoblastic cells. It was recently shown that FH can bind independently from and even compete with C1q for binding to apoptotic surfaces and other targets (25). When FH binds directly to the apoptotic surface and tissue debris, it may serve to further dampen the inflammatory response initiated via the classical pathway.

We found an increasing frequency of *C4A* deficiencies with the severity of the PE diagnosis. *C4A* deficiencies are observed in approximately 16% of the Finnish general population (14, 26). We found a 2.5-fold increase in the occurrence of *C4A* deficiency (40%) in the late-onset PE group and a slightly higher frequency in the early-onset group (43%). Because of the small sample size, the studies need to be repeated in a larger material. Based on our interesting preliminary results such a study is underway. While the two C4 proteins, C4A and C4B, have mainly overlapping functions, some differences may also be observed (27). *C4A* deficiencies are often associated with susceptibility to autoimmune diseases (28). Our patients and controls were selected so that patients with immunological diseases that might be associated with *C4A* deficiency, such as SLE, were excluded. Low plasma C4 levels in preeclamptic women have been previously found to be the only abnormality in a panel of C components measured in PE pregnancies and healthy controls (29). The presence of two C4 products with differing functions could explain, why in that study, C4 levels observed in the placenta did not associate with the known deficiency status, as the used antibody does not discriminate between the two forms of C4.

C4bp is a major soluble protein that binds to C4b and regulates the classical pathway C3 convertase. We observed C4bp binding typically and intensely to apoptotic fragments and structures including shed and knotted syncytium. Because of this, it was not possible to quantitatively differentiate if fluorescence intensity levels in relation to disease status were due to the different levels of physical damage of the placenta or to truly different levels of C4bp (30). C4bp is known to bind directly to surfaces of apoptotic and necrotic cells (31, 32). Furthermore, syncytial bodies such as syncytial knots and sprouts might be more common in PE (33). Interestingly, analyses of the high-intensity fluorescence areas showed a strong correlation between C4bp, C4, and C9. This could be indicative of classical pathway activation. Previous reports of C4bp in PE are few and inconclusive. Mellembakken et al. found no difference in C4bp plasma levels of preeclamptics and controls, while Schjetlein et al. reported a decrease in C4bp plasma levels with increasing severity of PE (29, 34). Recently, mutations in C4bp were found to be related to recurrent pregnancy loss in certain patients (10).

Factor H is the most important regulator of the alternative pathway. Generally, FH was observed in abundance in most of the placentae. The absence of STB staining for FH in the PE cases, a trend which was also apparent with other C components, is likely due to the loss of surface negative charge and/or disturbed structure of the STB in the preeclamptic placentae. In clusters of C3b deposition, e.g., in areas of STB damage, FH would also bind. Considering that FH is a soluble molecule, it seems atypical that in 83% of cases and 40% controls, intense FH deposition was observed in the stromas of the tissue. This suggests an increased requirement of the tissue for protection from C attack in majority of the placentae from preeclamptic pregnancies. Interestingly, FH is known to be produced extrahepatically in certain tissues, including the placenta (35). It is likely, that in the placentae with staining of the stroma, the observed FH profile is a heterogeneous combination of the maternal FH localized on the syncytium and fetal FH localized in the villar arterial endothelium and tissue stromas. At these sites, FH would regulate the alternative pathway.

C3 is the most central component of the C system. We found C3 abundantly in the placenta. In the high-intensity area analysis, C3b/iC3b deposition correlated with C1q and negatively with FH deposition in the early-onset PE patients. This suggests that in the most difficult cases of PE where, the alternative pathway has become activated and, as a result, C3 deposits were observed, regulation by FH had failed to protect the placenta. This relationship was missing in the late-onset PE and control groups. There were no clear differences in the intensity or distribution of C3d between placentas from PE patients and healthy controls. The kinetics of C3d deposition differs from that of C3b and iC3b. C3d is usually found as a remnant from prolonged complement activation, especially in basement membranes.

C3d was localized most clearly to the sub-syncytium basement membrane. There was no difference in the intensity of C3d expression between the control and PE groups. This is in contrast to Sinha et al., who observed that more C3d deposition occurred in the trophoblast basement membrane in PE than in normal placentae, and that the positivity was more marked in the severe PE group than in patients with mild PE (36). In C3-knockout mice, C3 has been found to be needed for successful pregnancy. C3-knockout mice had a lower rate of conception and the fetal reabsorption rate was higher, while the fetal and placental weights were lower in these mice (8). Furthermore, in mice the C3-regulator Crry was found to be crucial to a successful pregnancy (9).

While MCP is an important inhibitor of C activation in the healthy maternal–fetal interface, C4bp apparently has a different role in binding mainly to apoptotic structures and damaged STB. This was reflected by a negative correlation between C4bp and MCP intensity in the controls. The correlation between MCP and DAF (CD55), which was observed in the high-intensity areas of the control placentae showed that these regulators synergize each other in the regulation of C activation in the third trimester placenta. They are probably both needed for the control of C activation on the syncytium. Membrane-bound regulators of complement DAF, MCP, and CD59 have been observed in the healthy first trimester and term placentae, where they serve to protect the developing placenta from C attack (5, 37, 38). In general, no differences in the CD59 patterns or abundance were observed between the preeclamptics and controls.

Increased endoglin staining was observed in PE samples. Our results corroborate the findings of Sitras et al. and Nishizawa et al. who both found increased levels of endoglin in PE, but could not differentiate late- and early-onset patient groups with regard to endoglin expression (39, 40). s-eng is a placenta-derived TGF-beta co-receptor known to be elevated in PE although reports of circulating s-eng levels correlating with disease severity are not consistent (16). Since it is known that s-eng is more abundant in PE than in healthy pregnancies, we used it as a positive control to verify the ability of our methodology to detect changes in immunohistochemical stain pattern and intensity between PE and control placental samples.

## Conclusion

Here, we have described for the first time the expression of a large panel of C system components in the placentae of PE pregnancies. It is apparent that complement is involved in multiple ways in both normal pregnancy as well as in PE.

A notable difference between two soluble C regulators was observed. C4bp was found to bind directly to apoptotic syncytial structures while fetal FH apparently provides an overall, broad scale protection to the placental tissue.

Correlation of MAC deposition with the classical pathway activating components in the patient groups and negative correlation in the controls may be indicative of C regulation breakdown and/or uncontrolled classical pathway activation in PE.

In our small cohort partial *C4A* deficiency was more frequent in patients than controls. The high incidence of *C4A* deficiency in PE might bear functional importance and the issue needs to be examined in a larger material.

## Author Contributions

Anna Inkeri Lokki and Jenni Heikkinen-Eloranta did the laboratory work for the project, analyzed the data, and drafted the manuscript. Jenni Heikkinen-Eloranta described the patient material with help from Hannele Laivuori and Terhi Saisto. Hannele Laivuori is the head of the FINNPEC board and together with Terhi Saisto and Jenni Heikkinen-Eloranta she collected the samples and clinical data. Hanna Jarva and Seppo Meri are experts in the complement system and participated in planning the laboratory methodology (Hanna Jarva) and providing the materials and laboratory space (Seppo Meri). Marja-Liisa Lokki is a specialist in the field of MHC genetics and planned, analyzed, and helped to interpret the C4 genetic portion. Hannele Laivuori and Seppo Meri came up with the study question planned the project in collaboration with Anna Inkeri Lokki and Jenni Heikkinen-Eloranta.

## Conflict of Interest Statement

The authors declare that the research was conducted in the absence of any commercial or financial relationships that could be construed as a potential conflict of interest.
